# Unraveling the obesity paradox in small cell lung cancer immunotherapy: unveiling prognostic insights through body composition analysis

**DOI:** 10.3389/fimmu.2024.1439877

**Published:** 2024-08-26

**Authors:** Ruoxin Fang, Ling Yan, Sha Xu, Yuchen Xu, Tian Gan, Jun Gong, Junhong Zhang, Conghua Xie, Zhengkai Liao

**Affiliations:** ^1^ Department of Radiation and Medical Oncology, Zhongnan Hospital of Wuhan University, Hubei Key Laboratory of Tumor Biological Behaviors, Hubei Cancer Clinical Study Center, Wuhan, Hubei, China; ^2^ Department of Cardiology, Renmin Hospital of Wuhan University, Hubei Key Laboratory of Metabolic and Chronic Diseases, Wuhan, Hubei, China; ^3^ Department of Radiology, Zhongnan Hospital of Wuhan University, Wuhan, Hubei, China

**Keywords:** small cell lung cancer, immunotherapy, body mass index, sarcopenia, obesity

## Abstract

**Background:**

The advent of immunotherapy has changed the landscape of SCLC treatment, although the identification of reliable prognostic biomarkers remains a formidable challenge. Our objective was to investigate the prognostic implications of obesity and body composition in SCLC immunotherapy while seeking a straightforward anthropometric measure.

**Methods:**

This retrospective study analyzed data from patients with SCLC who underwent immunotherapy between 2019 and 2023. Body composition and waist circumference (WC) were analyzed using 3D slicer software on baseline CT images. Quantitative measures, including skeletal muscle index (SMI), total adipose tissue index (TATI), and other indicators at the L3 level, along with body shape index (BSI) and additional indicators based on WC, were obtained. The relationships between these indicators, response, PFS, OS, and their interconnections were examined.

**Results:**

A total of 145 SCLC patients who received immunotherapy were identified, of whom 133 met the inclusion criteria. In univariate analysis, a BMI≥28 kg/m^2^ was associated with a PFS advantage (HR 0.42, p=0.04), but this trend vanished in multivariate analysis. Body measurements exhibited stronger correlations with adipose tissue content, with BSI showing the highest correlation with muscle. In multivariate analysis, lower BSI was associated with poorer OS (HR 1.79, p=0.02). The association between muscle composition and prognosis was robust in univariate analysis but dissipated in multivariate analysis. However, accounting for a high TATI background significantly heightened the adverse effect of SMI on prognosis in the multivariate model.

**Conclusion:**

No clear association between BMI and SCLC immunotherapy prognosis was observed. However, high adiposity exacerbated the adverse effects of sarcopenia in SCLC immunotherapy, and BSI demonstrated potential as a straightforward prognostic measure.

## 1 Introduction

Small cell lung cancer (SCLC) represents a neuroendocrine tumor, comprising approximately 13%-15% of all lung cancers, and remains one of the most lethal malignancies. It is highly aggressive with a poor prognosis, and over 60% of patients are diagnosed in the extensive stage (1). Etoposide plus platinum is the standard treatment for SCLC. In the chemotherapy era, the median survival for SCLC was a mere 7 months, with a 2-year survival rate of 7-8% (2). Since 2019, immune checkpoint inhibitors (ICIs) have gained approval as the first-line treatment for extensive-stage small cell lung cancer (ES-SCLC). Although the addition of ICIs has significantly extended survival in SCLC patients, these improvements are underwhelming compared to those seen in non-small cell lung cancer (NSCLC), with ICIs extending median overall survival (OS) in SCLC by only 2-4.5 months (3). Nevertheless, there has been a notable increase in the number of patients surviving beyond 3 years compared to the chemotherapy (4). Identifying potential beneficiaries of ICIs in SCLC and intervening to enhance their benefits remain pressing challenges.

Recently, contrary to the adverse effects of obesity on tumor progression in many preclinical studies, several studies have suggested that obese or high body mass index (BMI) cancer patients may derive greater benefits from ICIs, which is called the “obesity paradox” (5). Research indicates that overweight and obese NSCLC patients undergoing ICIs exhibit better progression-free survival (PFS) and OS than their non-obese counterparts, with a more pronounced trend in patients expressing positive programmed cell death ligand-1 (PD-L1) (6). Similar trends have been observed in malignant melanoma (7, 8). However, some studies have failed to establish a link between BMI and the prognosis of immunotherapy (9, 10), highlighting BMI’s limitations as a measure that doesn’t capture specific body composition. Moreover, whether BMI is associated with the prognosis of immunotherapy for SCLC remains unexplored.

In recent years, there has been significant interest in obtaining specific body composition through computed tomography (CT) images. Multiple studies have highlighted the association between subcutaneous or visceral adipose tissue and the prognosis of immunotherapy (11, 12). Additionally, the evaluation of skeletal muscle at the L3 level is a well-established method for assessing sarcopenia (13). Chaunzwa et al. studied the impact of L3 level skeletal muscle and adipose tissue composition on the prognosis of advanced NSCLC immunotherapy using CT imaging, and found that a reduction in skeletal muscle content and an increase in the density of subcutaneous adipose tissue (SAT) were associated with worse prognosis (14). There are also some small-sample studies that have shown that CT-measured reductions in skeletal muscle are detrimental to the prognosis of immunotherapy for advanced NSCLC (15, 16). However, research into immunotherapy for SCLC is still quite scarce in this field. This study aims to investigate the correlation between body composition, as determined through CT imaging, and immunotherapy prognosis in ES-SCLC. In consideration of practical applicability, we have also incorporated new anthropometric indicators based on waist circumference (WC) with the hope of identifying a more suitable indicator than BMI to guide the management of ES-SCLC patients.

## 2 Methods

### 2.1 Patient population

We conducted a retrospective analysis of 145 patients with SCLC who underwent immune checkpoint inhibitor (ICI) therapy at Zhongnan Hospital of Wuhan University from April 2019 to April 2023. Inclusion criteria comprised pathologically confirmed SCLC, CT-confirmed extensive-stage disease, receipt of at least one anti-programmed cell death protein 1 (PD-1) or anti-PD-L1 treatment, and the availability of abdominal CT or positron emission tomography/computed tomography (PET/CT) within two months before or after the first immunotherapy. Exclusion criteria included lung adenocarcinoma transformation into small cell lung cancer (n=3), unknown baseline time of immunotherapy (n=7), and loss of follow-up (n=2). Ultimately, 133 patients were included in the study.

Clinical information, including gender, age, height, weight, Eastern Cooperative Oncology Group performance status (ECOG PS), stage, metastatic organs, ICI types, and previous treatment, was collected from electronic medical records. Response, PFS, and OS were obtained through electronic medical records and telephone follow-up. Response was evaluated based on RECIST V.1.1, with a patient considered to have achieved a response if they attained complete response (CR) or partial response (PR). Our study included 4 patients who achieved CR, while efficacy evaluation was not available for 11 patients. PFS was defined as the time from the treatment start to progression or death. OS was defined as the time from the treatment start to death or last follow-up.

### 2.2 Measurement of WC and body composition

**Figure 1:**
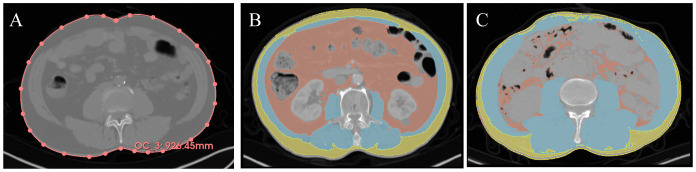
Representative imaging contour results. Yellow = SAT, Red = VAT, Blue = muscle. **(A)** Waist circumference measurement, the red line represents the waist circumference. **(B)** Representative low SMI + high TATI. **(C)** Representative high SMI + low TATI. SAT, subcutaneous adipose tissue; VAT, visceral adipose tissue; SMI, skeletal muscle index; TATI, total adipose tissue index.

Analysis of non-contrasted PET-CT or CT images was performed using 3D slicer (USA, Version 5.0.2) (17). The entire image file was uploaded to the software, and the L3-L4 level of the CT image was determined (**Figure 1**). Muscle tissue was defined with a threshold of -29 to +150 HU, SAT with a threshold of -190 to -30 HU, and visceral adipose tissue (VAT) with a threshold of -150 to -50 HU. Two researchers, trained in imaging, independently mapped each patient’s body composition at the L3 level and WC at the L3-L4 disc level. The area of each section and skeletal muscle density (SMD) were computed using the Segment Geometry plugin (18). Every image is verified by professional radiologist.

### 2.3 Determination of the cut-off value

BMI is calculated as weight (kg)/height (m)². To address variations in body shape specific to Asians and Caucasians (19), we adopted Chinese adult classifications: Normal < 24 kg/m², 24 kg/m² ≤ Overweight < 28 kg/m², and Obese ≥ 28 kg/m². WC is considered a superior indicator of central obesity, with high WC defined as ≥ 0.9m for males and ≥ 0.8m for females (20). Waist-to-Height Ratio (WHtR), representing the ratio of WC to height, is considered high when WHtR > 0.5. Other novel anthropometric indicators, namely Relative Fat Mass Index (RFM), Body Shape Index (BSI), Body Roundness Index (BRI), and Weight-Adjusted-Waist Index (WWI), provide a more nuanced reflection of body fat and total fat mass distribution (21). As there are no established reference boundaries for Asians presently, we classified them into quartiles (refer to **Supplementary Table S1** for formulas and boundary values).

Skeletal Muscle Index (SMI) is computed as muscle area (cm²)/height (m)². According to the international consensus on sarcopenia diagnosis (13), SMI<55 cm²/m² for males and SMI<39 cm²/m² for females defines sarcopenia. SMD, a measure of muscle attenuation associated with myosteatosis, was classified using quartiles. Skeletal Muscle Gauge (SMG), a composite index integrating SMI and SMD, is calculated as SMI multiplied by SMD and considered low when SMG<1475, as per Shachar et al.’s study (22, 23), Lean Body Mass (LBM) is estimated using the L3 muscle area (24), while VAT Index (VATi) and SAT Index (SATi) are standardized VAT and SAT areas, respectively, and Total Adipose Tissue Index (TATI) is the sum of VAT Index and SAT Index, all classified by quartiles.

### 2.4 Statistical analysis

Continuous variables were compared between groups using the student-t test or Mann-Whitney U test, and categorical variables were compared using the χ² test. PFS and OS were assessed using the Kaplan-Meier (KM) method, with group comparisons performed using the log-rank test. Univariate and multivariate Cox regression models were employed to estimate associations between BMI, anthropometric measures, and body composition with survival, adjusting for covariates such as age, gender, stage, ICI line, and ICI types. Logistic regression models were established to evaluate the association between each index and response incidence. Spearman correlation analysis was used to assess the correlation between various indicators. Interactions between TATI with SMI and SMG were calculated following Källberg et al.’s method (25). The main criterion for determining whether there is an interaction is based on the p-value and confidence interval (CI) of the interaction term (SMG×TATI or SMI×TATI), and stratified analysis was conducted in the multivariate model to control for variables. Statistical analyses were carried out using R V.4.2.2.

## 3 Results

### 3.1 Patient characteristics

A total of 133 patients were included in the analysis (**Table 1**), with the last follow-up date on October 1, 2023, and a median follow-up time of 552 days. At the last follow-up, 48 patients were still alive. The median PFS was 169 days, and the median OS was 331 days. Of the total, 62 patients achieved CR or PR, resulting in an Overall Response Rate (ORR) of 50.82%. The median time from baseline CT to immunotherapy initiation was 8 (IQR 4-24) days.

**Table 1:** Patient baseline characters.

	Overall (N=133)	Normal (N=82)	Overweight (N=40)	Obese (N=11)	P value
Age, Mean (SD)	63.1 (9.49)	63.0 (8.81)	64.4 (9.40)	59.4 (13.9)	0.288
Gender, n (%)					0.465
Male	114 (85.7%)	70 (85.4%)	33 (82.5%)	11 (100%)	
Female	19 (14.3%)	12 (14.6%)	7 (17.5%)	0 (0.00%)	
PS Score, n (%)					0.218
0-1	104 (78.2%)	60 (73.2%)	35 (87.5%)	9 (81.8%)	
≥2	29 (21.8%)	22 (26.8%)	5 (12.5%)	2 (18.2%)	
Clinical Stage, n (%)					0.873
III	21 (15.8%)	13 (15.9%)	7 (17.5%)	1 (9.09%)	
IV	112 (84.2%)	69 (84.1%)	33 (82.5%)	10 (90.9%)	
Metastatic Organs, n (%)					0.763
0	21 (15.8%)	13 (15.9%)	7 (17.5%)	1 (9.09%)	
1-2	65 (48.9%)	38 (46.3%)	22 (55.0%)	5 (45.5%)	
≥3	47 (35.3%)	31 (37.8%)	11 (27.5%)	5 (45.5%)	
ICI Line, n (%)					0.070
First line	93 (69.9%)	60 (73.2%)	23 (57.5%)	10 (90.9%)	
Second and posterior line	40 (30.1%)	22 (26.8%)	17 (42.5%)	1 (9.09%)	
ICI type, n (%)					0.419
PD-L1	61 (45.9%)	40 (48.8%)	15 (37.5%)	6 (54.5%)	
PD-1	72 (54.1%)	42 (51.2%)	25 (62.5%)	5 (45.5%)	
Waist Circumference, Mean (SD)	84.9 (11.2)	79.5 (9.30)	91.6 (6.86)	100 (8.57)	<0.001
BMI, Mean (SD)	23.0 (3.43)	20.8 (2.02)	25.7 (1.11)	29.4 (1.15)	<0.001
Sarcopenic, n (%)					<0.01
No	39 (29.3%)	18 (22.0%)	13 (32.5%)	8 (72.7%)	
Yes	94 (70.7%)	64 (78.0%)	27 (67.5%)	3 (27.3%)	

Patients had a median age of 64, with a majority being male (85.7%). The majority of patients were classified as stage IV (84.2%) based on TNM staging, and overall health was generally favorable. Chemo-immunotherapy was the predominant first-line treatment (69.9%), with a similar distribution between anti-PD-1 and anti-PD-L1 treatments. Sarcopenia was prevalent at baseline, affecting 70.7% of patients, with higher incidence observed in those with normal and overweight BMI compared to obese individuals.

### 3.2 Associations with BMI

We utilized the KM method to analyze survival differences among BMI subgroups (**Supplementary Figure S1**). Overall, no significant differences were observed in PFS and OS among the subgroups. However, in pairwise comparisons, PFS was significantly better in the obese group compared to the overweight group (p-value=0.04). In univariate analysis, the response in the overweight group was significantly worse than the normal group (OR 0.43, 95% CI 0.18 to 0.95, p-value=0.04), and the PFS in the obese group was significantly better than the overweight group (HR 0.42, 95% CI 0.19 to 0.96, p-value=0.04). However, these differences were not significant in multivariate analysis (**Supplementary Table S2**).

### 3.3 Correlation between each indicator

**Figure 2:**
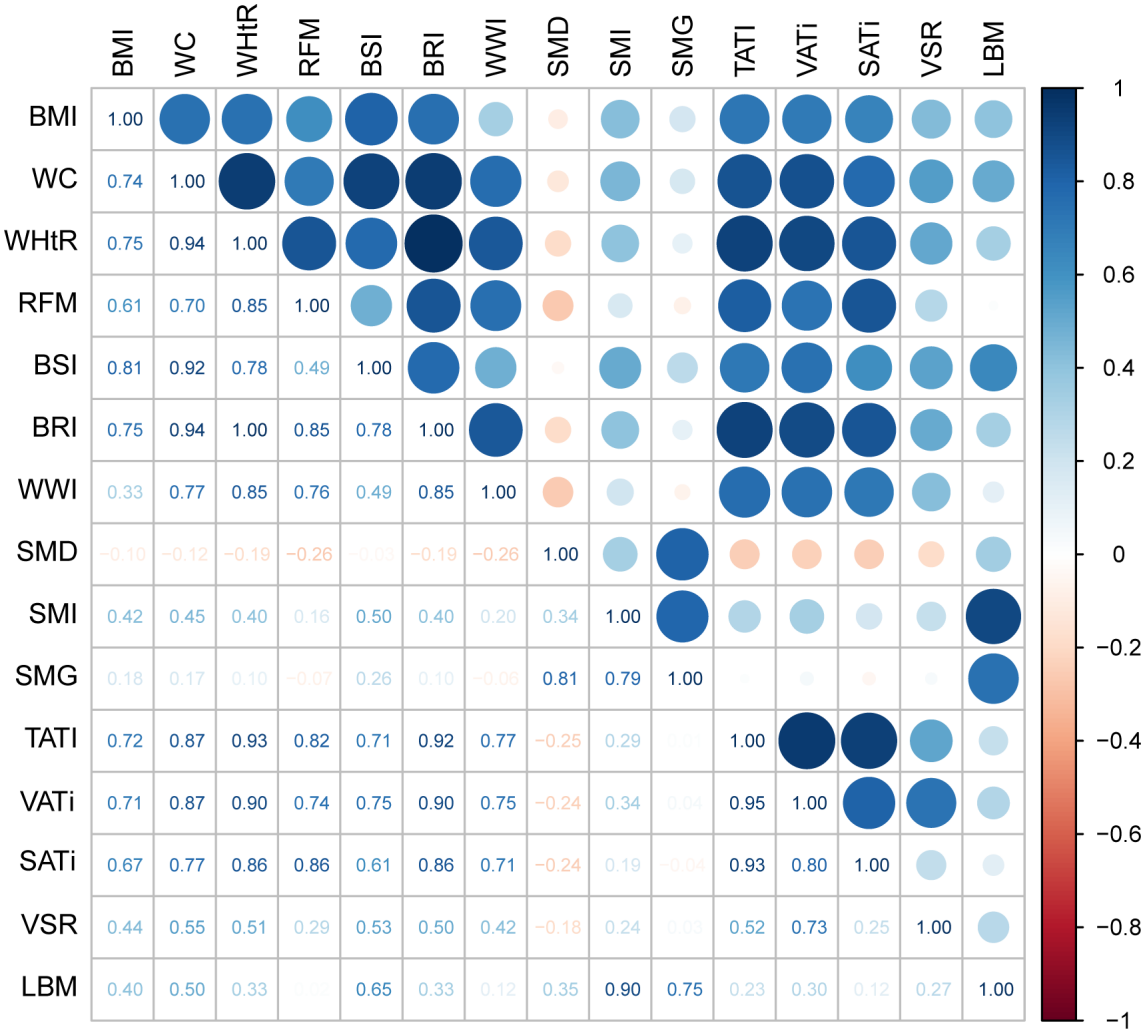
The heatmap showing the correlation between various anthropometric indicators and body composition. The numbers in the chart represent the correlation coefficient. A correlation coefficient>0.7 is considered a strong correlation, while 0.3<correlation coefficient ≤ 0.7 is considered a moderate correlation, and correlation coefficient<0.3 is considered a weak correlation. BMI, body mass index; WC, waist circumference; WHtR, waist-to-height ratio; RFM, relative fat mass index; BSI, body shape index; BRI, body roundness index; WWI, weight-adjusted-waist index; SMI, skeletal muscle index; SMD, skeletal muscle density; SMG, skeletal muscle gauge; TATI, total adipose tissue index; VATi, visceral adipose tissue index; SATi, subcutaneous adipose tissue index; VSR, visceral to subcutaneous adipose tissue area ratio; LBM, lean body mass.

Given the inclusion of numerous anthropometric indicators, in addition to commonly used BMI, in this study, other indicators also show potential for clinical application. We aimed to explore the correlation between these indicators and body composition (**Figure 2**). After excluding indicators with direct calculation relations, we observed that all anthropometric measures, including BMI, were strongly associated with adipose composition and weakly associated with muscle composition. The anthropometric indicator with the strongest correlation with muscle tissue is BSI, with a correlation coefficient of 0.50 with SMI and 0.65 with LBM. Additionally, BSI maintains a strong correlation with TATI (correlation coefficient 0.71).

### 3.4 Associations with anthropometric measures

As the clinical significance of new anthropometric indicators in cancer prognosis remains unclear, univariate and multivariate analyses were conducted with high quartile and low quartile as cut-off values, in addition to WC and WHtR. In univariate analysis, no significant associations were found between anthropometric measures and response, PFS, or OS. However, after adjusting for covariates, a poorer response was observed in patients with a lower WWI (OR 0.34, 95% CI 0.11 to 0.97, p-value=0.047), and a poorer OS was observed in patients with a lower BSI (HR 1.79, 95% CI 1.09 to 2.94, p-value=0.02) (**Supplementary Table S3**).

### 3.5 Associations with body composition measures

In univariate analysis, lower SMI and SMG were associated with worse response (SMI: OR 0.35, 95% CI 0.15 to 0.76, p-value=0.01; SMG: OR 0.4, 95% CI 0.18 to 0.84, p-value=0.02) and OS (SMI: HR 2.00, 95% CI 1.20 to 3.34, p-value=0.01; SMG: HR 1.63, 95% CI 1.06 to 2.51, p-value=0.03), and lower SMG was also associated with worse PFS (HR 1.73, 95% CI 1.18 to 2.55, p-value=0.01). Higher LBM was associated with better response (OR 2.95, 95% CI 1.29 to 7.17, p-value=0.01) and OS (HR 0.58, 95% CI 0.34 to 0.98, p-value=0.04). However, in multivariate analysis, none of these associations remained significant. Both higher (OR 0.29, 95% CI 0.10 to 0.80, p-value=0.02) and lower visceral to subcutaneous adipose tissue area ratio (VSR) (OR 0.20, 95% CI 0.05 to 0.70, p-value=0.01) were associated with poorer responses, suggesting that a moderate range of VSR may be more beneficial to treatment. Similar to new anthropometric indicators, univariate and multivariate analyses were conducted with high quartile and low quartile as cut-off values for VSR and LBM (**Table 2**).

**Table 2:** Univariate and multivariate analyses assess the association between body composition measures with response, PFS, and OS.

Univariate analysis			
Response (n=122)	OR	95% CI	P value
SMI (Sarcopenic VS Non-sarcopenic)	0.35	0.15 to 0.76	0.01*
SMD (Low VS High)	0.80	0.35 to 1.83	0.60
SMG (Low VS High)	0.40	0.18 to 0.84	0.02*
TATI (High VS Low)	0.96	0.42 to 2.17	0.92
VATi (High VS Low)	0.81	0.36 to 1.81	0.60
SATi (High VS Low)	1.24	0.55 to 2.84	0.61
VSR (Low VS High)	0.57	0.19 to 1.55	0.28
VSR (High VS Low)	0.52	0.22 to 1.18	0.12
LBM (Low VS High)	0.56	0.24 to 1.28	0.18
LBM (High VS Low)	2.95	1.29 to 7.17	0.01*
PFS (n=133)	HR	95% CI	P value
SMI (Sarcopenic VS Non-sarcopenic)	1.51	0.99 to 2.31	0.06
SMD (Low VS High)	1.46	0.96 to 2.21	0.08
SMG (Low VS High)	1.73	1.18 to 2.55	0.01*
TATI (High VS Low)	1.10	0.45 to 2.68	0.84
VATi (High VS Low)	0.71	0.45 to 1.13	0.15
SATi (High VS Low)	0.89	0.58 to 1.39	0.62
VSR (Low VS High)	1.07	0.64 to 1.79	0.81
VSR (High VS Low)	1.26	0.82 to 1.92	0.29
LBM (Low VS High)	1.16	0.75 to 1.80	0.50
LBM (High VS Low)	0.67	0.43 to 1.05	0.08
OS (n=133)	HR	95% CI	P value
SMI (Sarcopenic VS Non-sarcopenic)	2.00	1.20 to 3.34	0.01*
SMD (Low VS High)	1.47	0.93 to 2.32	0.10
SMG (Low VS High)	1.63	1.06 to 2.51	0.03*
TATI (High VS Low)	0.87	0.46 to 1.67	0.69
VATi (High VS Low)	0.68	0.40 to 1.14	0.14
SATi (High VS Low)	0.84	0.51 to 1.40	0.51
VSR (Low VS High)	0.99	0.55 to 1.78	0.97
VSR (High VS Low)	1.26	0.79 to 2.01	0.34
LBM (Low VS High)	1.55	0.97 to 2.48	0.07
LBM (High VS Low)	0.58	0.34 to 0.98	0.04*
Multivariable analysis			
Response (n=122)	OR	95% CI	P value
SMI (Sarcopenic VS Non-sarcopenic)	0.72	0.23 to 2.13	0.55
SMD (Low VS High)	1.14	0.39 to 3.47	0.81
SMG (Low VS High)	0.52	0.18 to 1.44	0.21
TATI (High VS Low)	1.08	0.37 to 3.13	0.88
VATi (High VS Low)	0.56	0.19 to 1.58	0.28
SATi (High VS Low)	1.32	0.46 to 3.98	0.62
VSR (Low VS High)	0.20	0.05 to 0.70	0.01*
VSR (High VS Low)	0.29	0.10 to 0.80	0.02*
LBM (Low VS High)	0.57	0.19 to 1.67	0.30
LBM (High VS Low)	1.25	0.43 to 3.73	0.69
PFS (n=133)	HR	95% CI	P value
SMI (Sarcopenic VS Non-sarcopenic)	1.11	0.68 to 1.81	0.67
SMD (Low VS High)	1.49	0.93 to 2.39	0.10
SMG (Low VS High)	1.54	0.99 to 2.41	0.06
TATI (High VS Low)	0.78	0.25 to 2.45	0.68
VATi (High VS Low)	0.70	0.44 to 1.12	0.14
SATi (High VS Low)	0.83	0.52 to 1.33	0.44
VSR (Low VS High)	1.14	0.67 to 1.96	0.63
VSR (High VS Low)	1.23	0.80 to 1.90	0.35
LBM (Low VS High)	1.17	0.74 to 1.84	0.50
LBM (High VS Low)	0.92	0.58 to 1.46	0.72
OS (n=133)	HR	95% CI	P value
SMI (Sarcopenic VS Non-sarcopenic)	1.40	0.77 to 2.54	0.27
SMD (Low VS High)	1.54	0.93 to 2.55	0.09
SMG (Low VS High)	1.55	0.97 to 2.49	0.07
TATI (High VS Low)	0.75	0.38 to 1.47	0.40
VATi (High VS Low)	0.69	0.41 to 1.18	0.18
SATi (High VS Low)	0.74	0.44 to 1.24	0.26
VSR (Low VS High)	1.21	0.66 to 2.22	0.54
VSR (High VS Low)	1.37	0.85 to 2.20	0.20
LBM (Low VS High)	1.36	0.83 to 2.23	0.22
LBM (High VS Low)	0.87	0.49 to 1.56	0.64

#Adjusted for age, gender, stage, ICI line and ICI types. *P ≤ 0.05.

OS, overall survival; PFS, progression free survival.

### 3.6 Interaction between SMI and TATI

**Figure 3:**
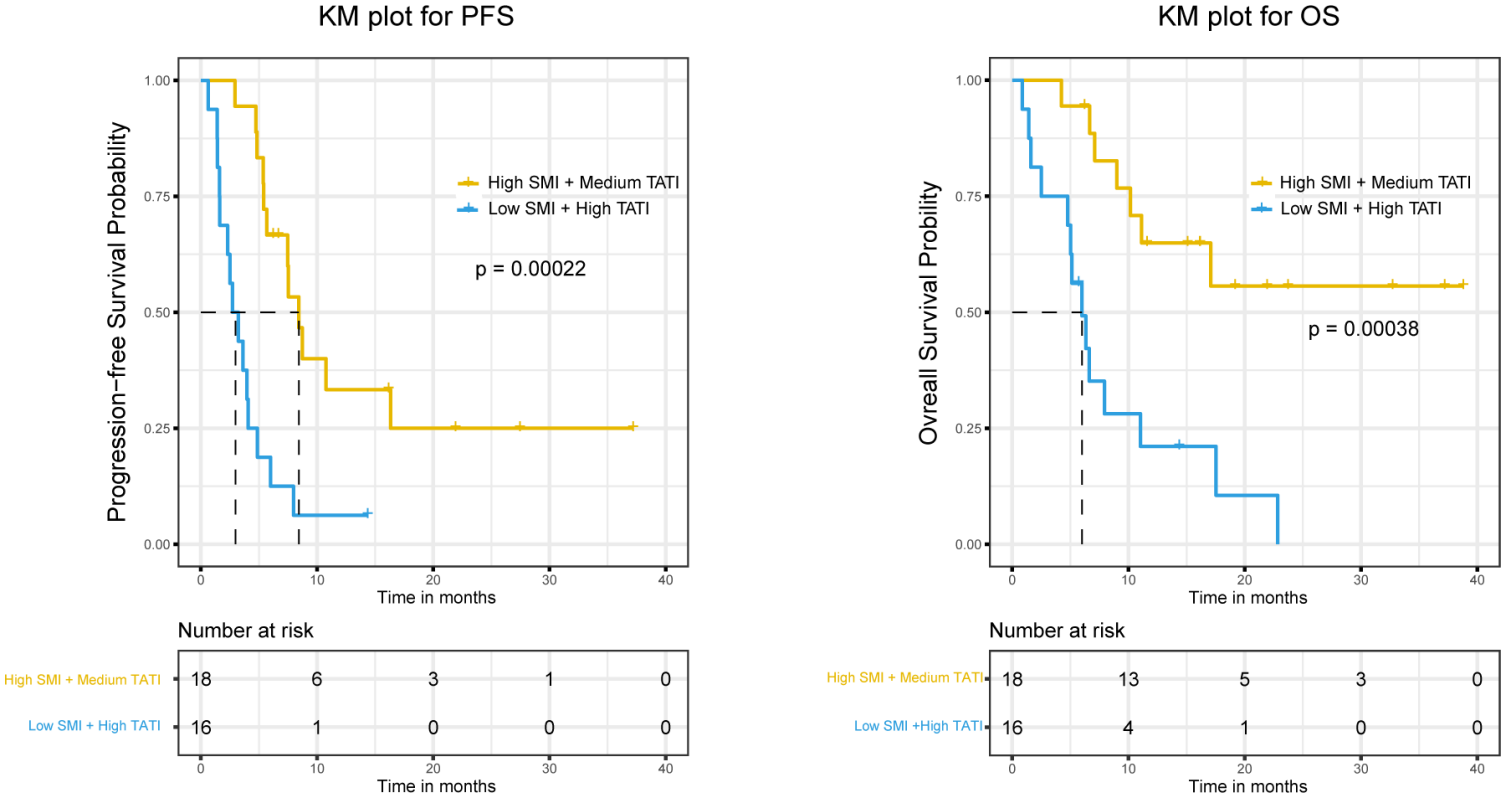
The Kaplan-Meier curves of PFS and OS were compared among different SMI and TATI combinations. SMI, skeletal muscle index; TATI, total adipose tissue index; PFS, progression free survival; OS, overall survival.

Examining potential interactions between muscle composition and adipose composition, we first explored the interaction between SMG and TATI, but no significant interaction was found (**Supplementary Tables S4, S5**). Despite the significant p-value, when combined with the CI and stratified analysis results, we do not find an interaction between SMG and TATI in our cohort. Subsequently, we examined the interaction between SMI and TATI, finding that the interaction term SMI × TATI was significant for PFS (HR 0.93, 95% CI 0.89 to 0.98, p-value=0.0034) but not for response and OS (**Supplementary Table S6**). Additionally, we explored whether there was an additive interaction between these variables, but no statistically significant additive interaction effect was found. Subsequently, we controlled SMI and TATI respectively in the multivariate analysis to assess whether the relationship between another indicator and response, PFS, and OS changed (**Table 3**). We observed that when high SMI was controlled, PFS significantly improved with high TATI (HR 0.39, 95% CI 0.16 to 0.99, p-value=0.046). Conversely, when high TATI was controlled, the negative impact of low SMI on PFS (HR 4.21, 95% CI 1.01 to 17.57, p-value=0.049) and OS (HR 10.96, 95% CI 2.36 to 50.90, p-value=0.0022) became significantly greater. Finally, we employed the KM method to examine survival differences among subgroups with different SMI and TATI combinations. Among all subgroups, the Low SMI + High TATI group exhibited the worst PFS and OS, with the largest difference observed when compared to the High SMI + Medium TATI group (**Figure 3**).

**Table 3:** Stratified analysis of the association between SMI and TATI with response, PFS, and OS.

Response (n=122)	OR	95% CI	P value
High SMI			
TATI (High VS Low)	1.04	0.76 to 1.44	0.79
Low SMI			
TATI (High VS Low)	0.92	0.70 to 1.19	0.51
High TATI			
SMI (Low VS High)	0.76	0.45 to 1.28	0.31
Low TATI			
SMI (Low VS High)	0.92	0.73 to 1.17	0.50
PFS (n=133)	HR	95% CI	P value
High SMI			
TATI (High VS Low)	0.39	0.16 to 0.99	0.049*
Low SMI			
TATI (High VS Low)	1.28	0.67 to 2.47	0.46
High TATI			
SMI (Low VS High)	4.21	1.01 to 17.57	0.05*
Low TATI			
SMI (Low VS High)	0.87	0.47 to 1.60	0.65
OS (n=133)	HR	95% CI	P value
High SMI			
TATI (High VS Low)	0.48	0.16 to 1.40	0.18
Low SMI			
TATI (High VS Low)	1.12	0.57 to 2.21	0.73
High TATI			
SMI (Low VS High)	10.96	2.36 to 50.90	<0.01**
Low TATI			
SMI (Low VS High)	1.16	0.55 to 2.42	0.70

Adjusted for age, gender, stage, ICI line and ICI types. *P ≤ 0.05; **P<0.01.

## 4 Discussion

To the best of our knowledge, this is the first study providing a comprehensive analysis of the association between body composition, anthropometric indexes, and the prognosis of immunotherapy in patients with ES-SCLC. In the era of chemotherapy, prior studies investigated the relationship between body composition, BMI, and the efficacy and prognosis of small-cell lung cancer (SCLC). While sarcopenia, diagnosed at the L3 levels on CT, has been linked to a poorer prognosis for SCLC (26, 27), the association with BMI remains uncertain. Some studies suggested a negative impact of low BMI on SCLC prognosis (28), while others reported complex and inconclusive associations, with trends toward better prognosis in patients with BMI >28kg/m² and weight loss (WL) ≤5% (29). There is a lack of evidence to suggest a link between WC and its newer variants and the prognosis of SCLC. In the era of immunotherapy, there is limited research, with only one pan-cancer study incorporating three SCLC patients receiving immunotherapy, yielding no conclusive results on BMI (30). Generally, SCLC has received less attention in anthropometric studies. As such the disease is lacking anthropometric biomarkers and presenting a significant clinical challenge.

In numerous preclinical studies, obesity has been correlated with tumor progression, attributed to its role in fostering a chronic inflammatory state and an immunosuppressive tumor immune microenvironment (31). Notably, obesity induces T-cell depletion, as evidenced by increased expression of PD-1, T cell immunoglobulin and mucin domain-3 (TIM-3), and lymphocyte activation gene-3 (LAG-3) in tumor-bearing mice with diet-induced obesity (DIO) (8, 32). However, the advent of immunotherapy has altered this scenario. ICIs counteract T-cell dysfunction by targeting PD-1 or PD-L1. Anti-PD-1 treatment for DIO mice in preclinical studies reversed immunosuppression in the tumor microenvironment (TME), with DIO mice exhibiting enhanced efficacy compared to the control group (33). Some clinical studies also indicated that patients with higher BMI derive more benefits from immunotherapy, termed the “obesity paradox” (6, 8). The debate around the “obesity paradox” centers on the evaluation index of BMI (5). Despite its clinical ubiquity, BMI is a relatively crude measure that inadequately reflects specific body composition. In our study, BMI demonstrated a weak correlation with muscle composition, while skeletal muscle has been established as a prognostic factor in various cancers (34). Recent studies have sought to elucidate the “obesity paradox” using imaging measurements. Young et al. (9), investigating the prognosis of immunotherapy for malignant melanoma, found no association between BMI and clinical outcomes, suggesting that the link between body composition and improved clinical outcomes is modest. Lee et al. (11), on the other hand, proposed that visceral fat might explain the “obesity paradox,” with its prognostic impact dependent on the systemic inflammatory state. Discrepancies between these studies may be attributed to differences in race and cut-off points. Although combining body composition and systemic immune-inflammation index (SII) is popular, establishing a causal relationship between the two remains debatable.

While our study focused on different populations and diseases, our findings generally align with those of Young et al. In univariate analysis and KM curves, the obese group exhibited advantages in terms of PFS and OS, but these advantages did not persist in multivariate analysis. Objectively, we did not identify a clear relationship between high BMI and the prognostic benefits of immunotherapy. Existing anthropometric measures, primarily based on height, weight, and waist circumference, are more closely tied to adipose content and less indicative of skeletal muscle. Among the examined anthropometric measures, BSI emerged as the most promising indicator, reflecting both skeletal muscle and adipose content. BSI was also associated with OS in multivariate analysis, though its efficacy as a biomarker requires further validation. Additionally, our study identified intriguing indicators, such as the association of WWI and LBM with response, potentially linked to the distribution of chemotherapeutic drugs (24). Both higher and lower VSRs were associated with worse responses, suggesting that a moderate VSR may confer better therapeutic benefits. Crucially, our data underscore the significance of the skeletal muscle-adipose tissue interaction. The detrimental effects of sarcopenia are significantly exacerbated in the presence of high adipose, consistent with the understanding that sarcopenic obesity portends worse outcomes (24, 35).This effect was more pronounced in SCLC than what Young et al. observed in malignant melanoma. Given SCLC’s neuroendocrine nature and diverse tumor syndromes, the interaction and crosstalk between tumor and non-tumor tissues merit consideration. Leptin concentration and the leptin/VAT ratio, indicative of adipokine influence, were associated with prolonged PFS in ES-SCLC patients (36). What’s more, anti-growth differentiation factor 15 (GDF-15) combined with anti-PD-1 therapy enhanced anti-PD-1 efficacy (37), as GDF-15 is closely tied to cachexia (38). Skeletal muscle and adipose, functioning as endocrine organs, engage in rich crosstalk in the body (39). Therapies targeting this interaction may not only address metabolic diseases but also enhance immunotherapy efficacy.

Several limitations must be acknowledged in our study. Primarily, being a single-center study introduces potential bias in population characteristics. Notably, our cohort exhibits a significant gender proportion bias, with over 80% of patients being male. The insufficient number of female patients precluded gender-stratified analysis. Moreover, many indicators lack clear-cut criteria, and employing quartiles to establish critical values may be inappropriate. Additional patient characteristics that could impact efficacy, such as immunotherapy-related adverse events and pretreatment weight loss, were not included. The relatively small sample size may affect statistical power, especially during further subgroup analyses. Findings regarding BMI require validation in a larger cohort.

## 5 Conclusion

In conclusion, our study did not reveal a clear association between BMI and the prognosis of SCLC immunotherapy. However, it reinforced the notion that a high-adipose background amplifies the adverse effects of sarcopenia in the context of SCLC immunotherapy. Notably, BSI emerged as a potential proxy for simple body composition assessment. Given the challenges in visually diagnosing sarcopenic obesity, our study underscores the importance of comprehensive nutritional assessment for cancer patients.

## Data Availability

The original contributions presented in the study are included in the article/**Supplementary Material**. Further inquiries can be directed to the corresponding authors.

## References

[1] Megyesfalvi Z Gay CM Popper H Pirker R Ostoros G Heeke S. Clinical insights into small cell lung cancer: Tumor heterogeneity, diagnosis, therapy, and future directions. CA Cancer J Clin. (2023) 73:620–52. doi: 10.3322/caac.21785 37329269

[2] Wang Q Gümüş ZH Colarossi C Memeo L Wang X Kong CY. SCLC: epidemiology, risk factors, genetic susceptibility, molecular pathology, screening, and early detection. J Thorac Oncol. (2023) 18:31–46. doi: 10.1016/j.jtho.2022.10.002 36243387 PMC10797993

[3] Iams WT Porter J Horn L. Immunotherapeutic approaches for small-cell lung cancer. Nat Rev Clin Oncol. (2020) 17:300–12. doi: 10.1038/s41571-019-0316-z PMC7212527 32055013

[4] Paz-Ares L Chen Y Reinmuth N Hotta K Trukhin D Statsenko G. Durvalumab, with or without tremelimumab, plus platinum-etoposide in first-line treatment of extensive-stage small-cell lung cancer: 3-year overall survival update from CASPIAN. ESMO Open. (2022) 7:100408. doi: 10.1016/j.esmoop.2022.100408 35279527 PMC9161394

[5] Farag KI Makkouk A Norian LA. Re-evaluating the effects of obesity on cancer immunotherapy outcomes in renal cancer: what do we really know? Front Immunol. (2021) 12:668494. doi: 10.3389/fimmu.2021.668494 34421889 PMC8374888

[6] Kichenadasse G Miners JO Mangoni AA Rowland A Hopkins AM Sorich MJ. Association between body mass index and overall survival with immune checkpoint inhibitor therapy for advanced non-small cell lung cancer. JAMA Oncol. (2020) 6:512–8. doi: 10.1001/jamaoncol.2019.5241 PMC6990855 31876896

[7] McQuade JL Daniel CR Hess KR Mak C Wang DY Rai RR. Association of body-mass index and outcomes in patients with metastatic melanoma treated with targeted therapy, immunotherapy, or chemotherapy: a retrospective, multicohort analysis. Lancet Oncol. (2018) 19:310–22. doi: 10.1016/s1470-2045(18)30078-0 PMC5840029 29449192

[8] Wang Z Aguilar EG Luna JI Dunai C Khuat LT Le CT. Paradoxical effects of obesity on T cell function during tumor progression and PD-1 checkpoint blockade. Nat Med. (2019) 25:141–51. doi: 10.1038/s41591-018-0221-5 PMC6324991 30420753

[9] Young AC Quach HT Song H Davis EJ Moslehi JJ Ye F. Impact of body composition on outcomes from anti-PD1 +/- anti-CTLA-4 treatment in melanoma. J Immunother Cancer. (2020) 8(2):e000821. doi: 10.1136/jitc-2020-000821 32747470 PMC7398101

[10] Donnelly D Bajaj S Yu J Hsu M Balar A Pavlick A. The complex relationship between body mass index and response to immune checkpoint inhibition in metastatic melanoma patients. J Immunother Cancer. (2019) 7:222. doi: 10.1186/s40425-019-0699-5 31426863 PMC6700794

[11] Lee JH Hyung S Lee J Choi SH. Visceral adiposity and systemic inflammation in the obesity paradox in patients with unresectable or metastatic melanoma undergoing immune checkpoint inhibitor therapy: a retrospective cohort study. J Immunother Cancer. (2022) 10(8):e005226. doi: 10.1136/jitc-2022-005226 36002189 PMC9413167

[12] He M Chen ZF Zhang L Gao X Chong X Li HS. Associations of subcutaneous fat area and Systemic Immune-inflammation Index with survival in patients with advanced gastric cancer receiving dual PD-1 and HER2 blockade. J Immunother Cancer. (2023) 11(6):e007054. doi: 10.1136/jitc-2023-007054 37349127 PMC10314655

[13] Fearon K Strasser F Anker SD Bosaeus I Bruera E Fainsinger RL. Definition and classification of cancer cachexia: an international consensus. Lancet Oncol. (2011) 12:489–95. doi: 10.1016/s1470-2045(10)70218-7 21296615

[14] Chaunzwa TL Qian JM Li Q Ricciuti B Nuernberg L Johnson JW. Body composition in advanced non-small cell lung cancer treated with immunotherapy. JAMA Oncol. (2024) 10:773–83. doi: 10.1001/jamaoncol.2024.1120 PMC11117154 38780929

[15] Nishioka N Naito T Notsu A Mori K Kodama H Miyawaki E. Unfavorable impact of decreased muscle quality on the efficacy of immunotherapy for advanced non-small cell lung cancer. Cancer Med. (2021) 10:247–56. doi: 10.1002/cam4.3631 PMC7826480 33300678

[16] Tsukagoshi M Yokobori T Yajima T Maeno T Shimizu K Mogi A. Skeletal muscle mass predicts the outcome of nivolumab treatment for non-small cell lung cancer. Med (Baltimore). (2020) 99:e19059. doi: 10.1097/md.0000000000019059 PMC7035054 32049805

[17] Fedorov A Beichel R Kalpathy-Cramer J Finet J Fillion-Robin JC Pujol S. 3D Slicer as an image computing platform for the Quantitative Imaging Network. Magn Reson Imaging. (2012) 30:1323–41. doi: 10.1016/j.mri.2012.05.001 PMC3466397 22770690

[18] Huie JM Summers AP Kawano SM. SegmentGeometry: A tool for measuring second moment of area in 3D slicer. Integr Org Biol. (2022) 4:obac009. doi: 10.1093/iob/obac009 35291672 PMC8919404

[19] WHO Expert Consultation. Appropriate body-mass index for Asian populations and its implications for policy and intervention strategies. Lancet. (2004) 363:157–63. doi: 10.1016/s0140-6736(03)15268-3 14726171

[20] Li Y Gui J Liu H Guo LL Li J Lei Y. Predicting metabolic syndrome by obesity- and lipid-related indices in mid-aged and elderly Chinese: a population-based cross-sectional study. Front Endocrinol (Lausanne). (2023) 14:1201132. doi: 10.3389/fendo.2023.1201132 37576971 PMC10419183

[21] Butt JH Petrie MC Jhund PS Sattar N Desai AS Køber L. Anthropometric measures and adverse outcomes in heart failure with reduced ejection fraction: revisiting the obesity paradox. Eur Heart J. (2023) 44:1136–53. doi: 10.1093/eurheartj/ehad083 PMC10111968 36944496

[22] Marquardt JP Roeland EJ Van Seventer EE Best TD Horick NK Nipp RD. Percentile-based averaging and skeletal muscle gauge improve body composition analysis: validation at multiple vertebral levels. J Cachexia Sarcopenia Muscle. (2022) 13:190–202. doi: 10.1002/jcsm.12848 34729952 PMC8818648

[23] Shachar SS Deal AM Weinberg M Williams GR Nyrop KA Popuri K. Body composition as a predictor of toxicity in patients receiving anthracycline and taxane-based chemotherapy for early-stage breast cancer. Clin Cancer Res. (2017) 23:3537–43. doi: 10.1158/1078-0432.Ccr-16-2266 PMC5511549 28143874

[24] Prado CM Lieffers JR McCargar LJ Reiman T Sawyer MB Martin L. Prevalence and clinical implications of sarcopenic obesity in patients with solid tumours of the respiratory and gastrointestinal tracts: a population-based study. Lancet Oncol. (2008) 9:629–35. doi: 10.1016/s1470-2045(08)70153-0 18539529

[25] Källberg H Ahlbom A Alfredsson L. Calculating measures of biological interaction using R. Eur J Epidemiol. (2006) 21:571–3. doi: 10.1007/s10654-006-9037-6 16969718

[26] Kim EY Kim YS Park I Ahn HK Cho EK Jeong YM. Prognostic significance of CT-determined sarcopenia in patients with small-cell lung cancer. J Thorac Oncol. (2015) 10:1795–9. doi: 10.1097/jto.0000000000000690 26484630

[27] Wang K Long W Sima X Zhao Y Xiao B Gulizeba H. Sarcopenia defined by skeletal muscle mass index at the third lumbar vertebra is a prognostic factor for extensive-stage small cell lung cancer patients: a retrospective study. J Thorac Dis. (2022) 14:2645–51. doi: 10.21037/jtd-22-782 PMC9344420 35928624

[28] Evcil FY Önal Ö Özkan EE. The effect of body mass index on survival in lung cancer. Nutr Cancer. (2023) 75:857–66. doi: 10.1080/01635581.2022.2159045 36573347

[29] Oswalt C Liu Y Pang H Le-Rademacher J Wang X Crawford J. Associations between body mass index, weight loss and overall survival in patients with advanced lung cancer. J Cachexia Sarcopenia Muscle. (2022) 13:2650–60. doi: 10.1002/jcsm.13095 PMC9745449 36268548

[30] Yeung C Kartolo A Holstead R Moffat GT Hanna L Hopman W. No association between BMI and immunotoxicity or clinical outcomes for immune checkpoint inhibitors. Immunotherapy. (2022) 14:765–76. doi: 10.2217/imt-2021-0250 35695057

[31] Quail DF Dannenberg AJ. The obese adipose tissue microenvironment in cancer development and progression. Nat Rev Endocrinol. (2019) 15:139–54. doi: 10.1038/s41574-018-0126-x PMC6374176 30459447

[32] Wu B Sun X Gupta HB Yuan B Li J Ge F. Adipose PD-L1 modulates PD-1/PD-L1 checkpoint blockade immunotherapy efficacy in breast cancer. Oncoimmunology. (2018) 7:e1500107. doi: 10.1080/2162402x.2018.1500107 30393583 PMC6209395

[33] Pingili AK Chaib M Sipe LM Miller EJ Teng B Sharma R. Immune checkpoint blockade reprograms systemic immune landscape and tumor microenvironment in obesity-associated breast cancer. Cell Rep. (2021) 35:109285. doi: 10.1016/j.celrep.2021.109285 34161764 PMC8574993

[34] Martin L Birdsell L Macdonald N Reiman T Clandinin MT McCargar LJ. Cancer cachexia in the age of obesity: skeletal muscle depletion is a powerful prognostic factor, independent of body mass index. J Clin Oncol. (2013) 31:1539–47. doi: 10.1200/jco.2012.45.2722 23530101

[35] Donini LM Busetto L Bischoff SC Cederholm T Ballesteros-Pomar MD Batsis JA. Definition and diagnostic criteria for sarcopenic obesity: ESPEN and EASO consensus statement. Clin Nutr. (2022) 41:990–1000. doi: 10.1016/j.clnu.2021.11.014 35227529

[36] Vita E Stefani A Piro G Mastrantoni L Cintoni M Cicchetti G. Leptin-mediated meta-inflammation may provide survival benefit in patients receiving maintenance immunotherapy for extensive-stage small cell lung cancer (ES-SCLC). Cancer Immunol Immunother. (2023) 72:3803–12. doi: 10.1007/s00262-023-03533-0 PMC10576666 37668709

[37] Haake M Haack B Schäfer T Harter PN Mattavelli G Eiring P. Tumor-derived GDF-15 blocks LFA-1 dependent T cell recruitment and suppresses responses to anti-PD-1 treatment. Nat Commun. (2023) 14:4253. doi: 10.1038/s41467-023-39817-3 37474523 PMC10359308

[38] Breit SN Brown DA Tsai VW. The GDF15-GFRAL pathway in health and metabolic disease: friend or foe? Annu Rev Physiol. (2021) 83:127–51. doi: 10.1146/annurev-physiol-022020-045449 33228454

[39] Guo L Quan M Pang W Yin Y Li F. Cytokines and exosomal miRNAs in skeletal muscle-adipose crosstalk. Trends Endocrinol Metab. (2023) 34:666–81. doi: 10.1016/j.tem.2023.07.006 37599201

