# Supplementary figures and images for: Unraveling the obesity paradox in small cell lung cancer immunotherapy: unveiling prognostic insights through body composition analysis
Source: Front Immunol. 2024 Aug 26;15:1439877. doi: 10.3389/fimmu.2024.1439877 (PMC11381398; doi:10.3389/fimmu.2024.1439877)

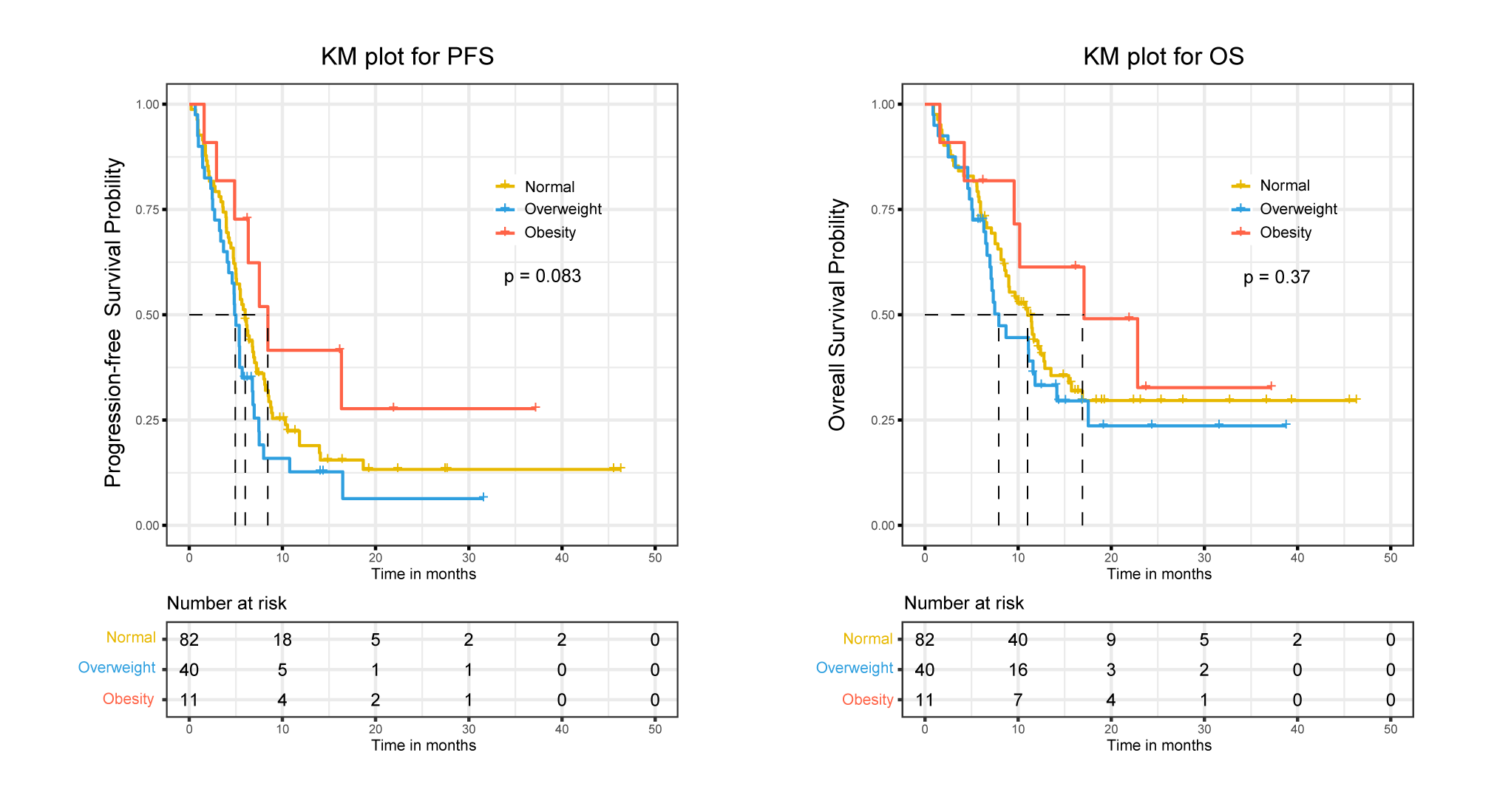

Supplement: Supplementary file 1 [file Image1.tif]
