# Supplementary material for: Unraveling the obesity paradox in small cell lung cancer immunotherapy: unveiling prognostic insights through body composition analysis
Source: Front Immunol. 2024 Aug 26;15:1439877. doi: 10.3389/fimmu.2024.1439877 (PMC11381398; doi:10.3389/fimmu.2024.1439877)
Supplement: Supplementary file 2 [file Table1.docx]

Table S1 | Quartiles and formula of each indicator.

| Indicator | Formula^#^ | Lower Quartiles | Upper Quartiles |
| --- | --- | --- | --- |
| RFM | 64-(20×height/WC)+12×sex, sex=0(men) and =1(women) | 20.39 | 29.35 |
| BSI | WC×weight^2/3^×height^5/6^ | 17.58 | 25.24 |
| BRI | 364.2-365.5×(1-((0.5×WC/π)^2^/(0.5×height)^2^))^0.5^ | 51.95 | 63.02 |
| WWI | (WC×100)/(weight^0.5^) | 9.86 | 11.07 |
| SMD (HU) | - | 30.35 | 37.10 |
| TATI | VAT index + SAT index |  |  |
| Male |  | 46.27 | 128.07 |
| Female |  | 85.82 | 134.33 |
| VAT index | VAT area (cm^2^)/height^2^ |  |  |
| Male |  | 17.13 | 63.68 |
| Female |  | 29.56 | 62.48 |
| SAT index | SAT area (cm^2^)/height^2^ |  |  |
| Male |  | 27.33 | 57.85 |
| Female |  | 53.83 | 79.18 |
| VSR | VAT area (cm^2^)/SAT area (cm^2^) |  |  |
| Male |  | 0.64 | 1.17 |
| Female |  | 0.55 | 0.83 |
| LBM | 0.3×(muscle area[cm²])+6.06 |  |  |
| Male |  | 43.40 | 54.05 |
| Female |  | 34.18 | 38.73 |

#Height (m); WC, waist circumference (m); Weight (kg).
