# Supplementary material for: Unraveling the obesity paradox in small cell lung cancer immunotherapy: unveiling prognostic insights through body composition analysis
Source: Front Immunol. 2024 Aug 26;15:1439877. doi: 10.3389/fimmu.2024.1439877 (PMC11381398; doi:10.3389/fimmu.2024.1439877)
Supplement: Supplementary file 3 [file Table2.docx]

Table S2 | Univariate and multivariate analyses assess the association between BMI with response, PFS, and OS.

| **Univariable analysis** |  |  |  |
| --- | --- | --- | --- |
| **Response (n=122)** | **OR** | **95%CI** | **P value** |
| Overweight VS Normal | 0.43 | 0.18 to 0.95 | 0.04* |
| Obese VS Normal | 1.83 | 0.47 to 9.01 | 0.41 |
| Obese VS Overweight | 4.31 | 1.01 to 22.73 | 0.06 |
| **PFS (n=133)** | **HR** | **95%CI** | **P value** |
| Overweight VS Normal | 1.36 | 0.90 to 2.07 | 0.14 |
| Obese VS Normal | 0.58 | 0.27 to 1.26 | 0.17 |
| Obese VS Overweight | 0.42 | 0.19 to 0.96 | 0.04* |
| **OS (n=133)** | **HR** | **95%CI** | **P value** |
| Overweight VS Normal | 1.23 | 0.77 to 1.96 | 0.39 |
| Obese VS Normal | 0.67 | 0.29 to 1.57 | 0.36 |
| Obese VS Overweight | 0.55 | 0.23 to 1.33 | 0.18 |
| **Multivariable analysis**^#^ |  |  |  |
| **Response (n=122)** | **OR** | **95%CI** | **P value** |
| Overweight VS Normal | 0.58 | 0.21 to 1.60 | 0.29 |
| Obese VS Normal | 0.84 | 0.17 to 4.87 | 0.83 |
| Obese VS Overweight | 1.45 | 0.26 to 9.52 | 0.68 |
| **PFS (n=133)** | **HR** | **95%CI** | **P value** |
| Overweight VS Normal | 0.93 | 0.59 to 1.48 | 0.76 |
| Obese VS Normal | 0.76 | 0.34 to 1.68 | 0.50 |
| Obese VS Overweight | 0.82 | 0.34 to 1.98 | 0.65 |
| **OS (n=133)** | **HR** | **95%CI** | **P value** |
| Overweight VS Normal | 0.81 | 0.49 to 1.33 | 0.40 |
| Obese VS Normal | 0.84 | 0.35 to 1.99 | 0.69 |
| Obese VS Overweight | 1.04 | 0.40 to 2.69 | 0.94 |

#Adjusted for age, gender, stage, ICI line and ICI types. *P≤0.05.

OS, overall survival; PFS, progression free survival.
