# Supplementary material for: Unraveling the obesity paradox in small cell lung cancer immunotherapy: unveiling prognostic insights through body composition analysis
Source: Front Immunol. 2024 Aug 26;15:1439877. doi: 10.3389/fimmu.2024.1439877 (PMC11381398; doi:10.3389/fimmu.2024.1439877)
Supplement: Supplementary file 4 [file Table3.docx]

Table S3 | Univariate and multivariate analyses assess the association between WC and its derived indicators with response, PFS, and OS.

| **Univariable analysis** |  |  |  |
| --- | --- | --- | --- |
| **Response (n=122)** | **OR** | **95%CI** | **P value** |
| WC (High VS Low) | 1.24 | 0.60 to 2.57 | 0.56 |
| WHtR (High VS Low) | 0.94 | 0.46 to 1.91 | 0.86 |
| RFM (High VS Low) | 1.13 | 0.50 to 2.56 | 0.76 |
| BSI (High VS Low) | 1.13 | 0.50 to 2.56 | 0.76 |
| BRI (High VS Low) | 0.88 | 0.39 to 1.99 | 0.75 |
| WWI (High VS Low) | 1.24 | 0.55 to 2.84 | 0.61 |
| RFM (Low VS High) | 0.63 | 0.28 to 1.40 | 0.26 |
| BSI (Low VS High) | 0.74 | 0.32 to 1.67 | 0.47 |
| BRI (Low VS High) | 0.74 | 0.33 to 1.66 | 0.47 |
| WWI (Low VS High) | 0.52 | 0.22 to 1.18 | 0.12 |
| **PFS (n=133)** | **HR** | **95%CI** | **P value** |
| WC (High VS Low) | 1.05 | 0.71 to 1.55 | 0.79 |
| WHtR (High VS Low) | 1.12 | 0.77 to 1.64 | 0.56 |
| RFM (High VS Low) | 0.90 | 0.58 to 1.40 | 0.65 |
| BSI (High VS Low) | 0.84 | 0.53 to 1.31 | 0.43 |
| BRI (High VS Low) | 0.95 | 0.61 to 1.47 | 0.81 |
| WWI (High VS Low) | 0.96 | 0.62 to 1.49 | 0.86 |
| RFM (Low VS High) | 0.91 | 0.58 to 1.42 | 0.68 |
| BSI (Low VS High) | 1.00 | 0.64 to 1.55 | 0.99 |
| BRI (Low VS High) | 0.79 | 0.51 to 1.24 | 0.31 |
| WWI (Low VS High) | 0.96 | 0.62 to 1.48 | 0.85 |
| **OS (n=133)** | **HR** | **95%CI** | **P value** |
| WC (High VS Low) | 0.77 | 0.49 to 1.20 | 0.24 |
| WHtR (High VS Low) | 0.84 | 0.55 to 1.29 | 0.42 |
| RFM (High VS Low) | 0.81 | 0.49 to 1.32 | 0.39 |
| BSI (High VS Low) | 0.85 | 0.51 to 1.40 | 0.52 |
| BRI (High VS Low) | 0.89 | 0.54 to 1.47 | 0.64 |
| WWI (High VS Low) | 0.80 | 0.48 to 1.32 | 0.38 |
| RFM (Low VS High) | 1.32 | 0.82 to 2.12 | 0.26 |
| BSI (Low VS High) | 1.46 | 0.91 to 2.35 | 0.12 |
| BRI (Low VS High) | 1.16 | 0.72 to 1.87 | 0.55 |
| WWI (Low VS High) | 1.29 | 0.80 to 2.07 | 0.30 |
| **Multivariable analysis**^#^ |  |  |  |
| **Response (n=122)** | **OR** | **95%CI** | **P value** |
| WC (High VS Low) | 1.53 | 0.60 to 4.05 | 0.38 |
| WHtR (High VS Low) | 0.95 | 0.39 to 2.36 | 0.91 |
| RFM (High VS Low) | 1.19 | 0.25 to 6.21 | 0.83 |
| BSI (High VS Low) | 1.06 | 0.37 to 3.16 | 0.91 |
| BRI (High VS Low) | 0.74 | 0.26 to 2.13 | 0.58 |
| WWI (High VS Low) | 1.11 | 0.39 to 3.30 | 0.84 |
| RFM (Low VS High) | 0.59 | 0.21 to 1.63 | 0.31 |
| BSI (Low VS High) | 0.62 | 0.22 to 1.70 | 0.35 |
| BRI (Low VS High) | 0.74 | 0.27 to 2.04 | 0.56 |
| WWI (Low VS High) | 0.34 | 0.11 to 0.97 | 0.047* |
| **PFS (n=133)** | **HR** | **95%CI** | **P value** |
| WC (High VS Low) | 1.03 | 0.68 to 1.55 | 0.90 |
| WHtR (High VS Low) | 1.07 | 0.72 to 1.59 | 0.75 |
| RFM (High VS Low) | 1.08 | 0.57 to 2.07 | 0.81 |
| BSI (High VS Low) | 0.90 | 0.56 to 1.44 | 0.65 |
| BRI (High VS Low) | 1.11 | 0.70 to 1.74 | 0.66 |
| WWI (High VS Low) | 1.27 | 0.80 to 2.04 | 0.31 |
| RFM (Low VS High) | 0.83 | 0.52 to 1.33 | 0.43 |
| BSI (Low VS High) | 1.15 | 0.72 to 1.82 | 0.57 |
| BRI (Low VS High) | 0.75 | 0.47 to 1.19 | 0.22 |
| WWI (Low VS High) | 0.91 | 0.58 to 1.43 | 0.68 |
| **OS (n=133)** | **HR** | **95%CI** | **P value** |
| WC (High VS Low) | 0.75 | 0.47 to 1.20 | 0.24 |
| WHtR (High VS Low) | 0.80 | 0.51 to 1.24 | 0.31 |
| RFM (High VS Low) | 1.16 | 0.59 to 2.28 | 0.66 |
| BSI (High VS Low) | 0.84 | 0.50 to 1.41 | 0.50 |
| BRI (High VS Low) | 1.05 | 0.62 to 1.76 | 0.86 |
| WWI (High VS Low) | 1.04 | 0.61 to 1.77 | 0.88 |
| RFM (Low VS High) | 1.24 | 0.75 to 2.05 | 0.40 |
| BSI (Low VS High) | 1.79 | 1.09 to 2.94 | 0.02* |
| BRI (Low VS High) | 1.12 | 0.68 to 1.85 | 0.65 |
| WWI (Low VS High) | 1.24 | 0.75 to 2.02 | 0.40 |

#Adjusted for age, gender, stage, ICI line and ICI types. *P≤0.05.

OS, overall survival; PFS, progression free survival.
