# Supplementary material for: Unraveling the obesity paradox in small cell lung cancer immunotherapy: unveiling prognostic insights through body composition analysis
Source: Front Immunol. 2024 Aug 26;15:1439877. doi: 10.3389/fimmu.2024.1439877 (PMC11381398; doi:10.3389/fimmu.2024.1439877)
Supplement: Supplementary file 5 [file Table4.docx]

Table S4 | Multiplication interactive effect of SMG and TATI.

| **Interaction items** | **OR** | **95%CI** | **P value** |
| --- | --- | --- | --- |
| **Response (n=122)** |  |  |  |
| SMG | 1.00 | 1.00 to 1.00 | 0.05* |
| TATI (High VS Low) | 0.93 | 0.03 to 19.19 | 0.96 |
| SMG × TATI | 1.00 | 1.00 to 1.00 | 0.98 |
| **PFS (n=133)** | **HR** | **95%CI** | **P value** |
| SMG | 1.00 | 1.00 to 1.00 | 0.62 |
| TATI (High VS Low) | 6.43 | 1.50 to 27.56 | 0.01* |
| SMG × TATI | 1.00 | 1.00 to 1.00 | 0.02* |
| **OS (n=133)** | **HR** | **95%CI** | **P value** |
| SMG | 1.00 | 1.00 to 1.00 | 0.20 |
| TATI (High VS Low) | 1.63 | 0.35 to 7.57 | 0.53 |
| SMG × TATI | 1.00 | 1.00 to 1.00 | 0.49 |

*P≤0.05. OS, overall survival; PFS, progression free survival.
