# Supplementary material for: Unraveling the obesity paradox in small cell lung cancer immunotherapy: unveiling prognostic insights through body composition analysis
Source: Front Immunol. 2024 Aug 26;15:1439877. doi: 10.3389/fimmu.2024.1439877 (PMC11381398; doi:10.3389/fimmu.2024.1439877)
Supplement: Supplementary file 6 [file Table5.docx]

Table S5 | Stratified analysis of the association between SMG and TATI with response, PFS, and OS.

| **Interaction items** | **OR** | **95%CI** | **P value** |
| --- | --- | --- | --- |
| **Response (n=122)** |  |  |  |
| High SMG |  |  |  |
| TATI (High VS Low) | 0.90 | 0.72 to 1.11 | 0.32 |
| Low SMG |  |  |  |
| TATI (High VS Low) | 1.15 | 0.84 to 1.58 | 0.39 |
| High TATI |  |  |  |
| SMG (Low VS High) | 1.02 | 0.67 to 1.53 | 0.94 |
| Low TATI |  |  |  |
| SMG (Low VS High) | 0.87 | 0.71 to 1.05 | 0.15 |
| **PFS (n=133)** | **HR** | **95%CI** | **P value** |
| High SMG |  |  |  |
| TATI (High VS Low) | 0.74 | 0.40 to 1.39 | 0.35 |
| Low SMG |  |  |  |
| TATI (High VS Low) | 1.47 | 0.66 to 3.28 | 0.35 |
| High TATI |  |  |  |
| SMG (Low VS High) | 2.33 | 0.75 to 7.21 | 0.14 |
| Low TATI |  |  |  |
| SMG (Low VS High) | 1.31 | 0.78 to 2.21 | 0.30 |
| **OS (n=133)** | **HR** | **95%CI** | **P value** |
| High SMG |  |  |  |
| TATI (High VS Low) | 0.69 | 0.34 to 1.40 | 0.30 |
| Low SMG |  |  |  |
| TATI (High VS Low) | 0.99 | 0.42 to 2.32 | 0.98 |
| High TATI |  |  |  |
| SMG (Low VS High) | 2.97 | 0.87 to 10.09 | 0.08 |
| Low TATI |  |  |  |
| SMG (Low VS High) | 1.38 | 0.81 to 2.37 | 0.24 |

Adjusted for age, gender, stage, ICI line and ICI types.
