# Supplementary material for: Unraveling the obesity paradox in small cell lung cancer immunotherapy: unveiling prognostic insights through body composition analysis
Source: Front Immunol. 2024 Aug 26;15:1439877. doi: 10.3389/fimmu.2024.1439877 (PMC11381398; doi:10.3389/fimmu.2024.1439877)
Supplement: Supplementary file 7 [file Table6.docx]

Table S6 | Multiplication interactive effect of SMI and TATI.

| **Interaction items** | **OR** | **95%CI** | **P value** |
| --- | --- | --- | --- |
| **Response (n=122)** |  |  |  |
| SMI | 1.01 | 0.99 to 1.04 | 0.35 |
| TATI (High VS Low) | 1.00 | 0.99 to 1.01 | 0.97 |
| SMI × TATI | 1.00 | 1.00 to 1.00 | 0.99 |
| **PFS (n=133)** | **HR** | **95%CI** | **P value** |
| SMI | 1.00 | 0.97 to 1.03 | 0.84 |
| TATI (High VS Low) | 47.50 | 4.26 to 530.14 | ＜0.01** |
| SMI × TATI | 0.93 | 0.89 to 0.98 | ＜0.01** |
| **OS (n=133)** | **HR** | **95%CI** | **P value** |
| SMI | 0.99 | 0.96 to 1.03 | 0.70 |
| TATI (High VS Low) | 10.66 | 0.79 to 143.61 | 0.07 |
| SMI × TATI | 0.96 | 0.91 to 1.01 | 0.09 |

**P＜0.01. OS, overall survival; PFS, progression free survival.
